# Theory‐based behavior change intervention to increase uptake of risk‐reducing salpingo‐oophorectomy in women with a *BRCA1* or *BRCA2* pathogenic variant: The PREVENT randomized controlled trial

**DOI:** 10.1002/cam4.6417

**Published:** 2023-08-21

**Authors:** Kelly A. Metcalfe, Tuya Pal, Steven A. Narod, Susan Armel, Salma Shickh, Kathleen Buckley, Scott T. Walters, Sarah Brennenstuhl, Anita Y. Kinney

**Affiliations:** ^1^ Lawrence S. Bloomberg Faculty of Nursing University of Toronto Toronto Ontario Canada; ^2^ Women's College Research Institute Toronto Ontario Canada; ^3^ Vanderbilt‐Ingram Cancer Center at the Vanderbilt University Medical Center Nashville Tennessee USA; ^4^ Princess Margaret Cancer Centre Toronto Ontario Canada; ^5^ Institute of Health Policy, Management and Evaluation University of Toronto Toronto Ontario Canada; ^6^ Grand River Hospital Kitchener Ontario Canada; ^7^ University of North Texas Health Science Center Fort Worth Texas USA; ^8^ Rutgers Cancer Institute of New Jersey New Brunswick New Jersey USA; ^9^ Department of Biostatistics and Epidemiology, School of Public Health Rutgers University Piscataway New Jersey USA

**Keywords:** BRCA1, BRCA2, cancer genetics, cancer prevention, clinical management

## Abstract

**Objective:**

To evaluate the effect of a theory‐based behavioral intervention delivered by genetic counselors on the uptake of risk‐reducing salpingo‐oophorectomy (RRSO) at 12 and 24 months by women with a *BRCA1* or *BRCA2* pathogenic variant (PV) compared to women who received usual care.

**Methods:**

In this two‐arm, multi‐site randomized controlled trial participants were randomized to receive a theoretically‐guided behavioral telephone intervention or usual care. Outcome data were collected at 12 and 24 months. Participants in the usual care arm were offered the intervention after 12 months.

**Results:**

Data on 107 participants were included in the analysis. There was no significant difference in the proportion of women who had a RRSO by 1 year (28.6%‐ intervention; 22.9%‐ usual care (*p* = 0.54)). At 1 year, women who received the intervention had significantly lower mean decisional conflict (*p*_interaction_ <0.001) and a higher mean knowledge score at one‐year compared to usual care (*p*_interaction_ <0.001). At 2 years, 53.9% of participants in the intervention arm had RRSO compared to 32.6% in usual care (*p* = 0.05).

**Conclusions:**

A theory‐based behavioral intervention delivered by genetic counselors to women with a *BRCA* PV who chose not to have the recommended RRSO was effective at reducing decisional conflict and increasing knowledge in women with a *BRCA1* or *BRCA2* PV.

## 1 INTRODUCTION

Women with a pathogenic or likely pathogenic variant (PV) in *BRCA1* or *BRCA2* have high lifetime risks of developing ovarian cancer. The cumulative risk of ovarian cancer to age 80 years for women with a *BRCA1* PV is 44%, and for *BRCA2* is 17%.^1^ This can be compared to a 1% risk of ovarian cancer in the general population.^2^ For women with a *BRCA* mutation, the 10‐year survival rate for clinically detected ovarian cancer is only 35%.^3^ As a result, managing the risk of ovarian cancer is critical for women who have a PV in *BRCA1* or *BRCA2*.

Risk‐reducing salpingo‐oophorectomy (RRSO) is the removal of the ovaries and fallopian tubes. In women with a PV in *BRCA1* or *BRCA2*, this preventive surgery reduces the risk of ovarian/peritoneal/fallopian tube cancer by 80%, and reduces the risk of all‐cause mortality by 77%.^3^ Ovarian screening with transvaginal ultrasound is not effective in reducing the risk of death from ovarian cancer. When comparing screening ultrasound to RRSO in women with a *BRCA1* PV, RRSO offers a 76% reduction in mortality.^4^

Currently, the National Comprehensive Cancer Network (NCCN) recommends RRSO for management of ovarian cancer risk in women with a *BRCA* PV. For women with a *BRCA1* PV, the recommended age for preventive surgery is between 35 and 40 years, and for *BRCA2* is 40–45 years. However, uptake of RRSO in these high‐risk women is not ideal. In a large international study of 6223 women with a *BRCA1* or *BRCA2* PV from 10 countries, we reported a RRSO uptake rate of 65%.^5^ This low uptake rate may be a result of our current models of genetic testing, which may or may not include post‐test genetic counseling and ongoing follow‐up of women with a *BRCA1* or *BRCA2* PV. It is unclear if follow‐up genetic or behavioral counseling would increase uptake of RRSO in this population.

Kinney et al. (2014) have previously reported on a randomized controlled trial (TeleCARE) conducted in the United States to evaluate the effect of a remote risk communication and behavior change intervention among individuals at a high‐risk of developing colorectal cancer based on family history.^6^ They compared the efficacy of a theory based telephone psychoeducational intervention delivered by genetic counselors with a mailed educational brochure for improving uptake of colonoscopy screening among at‐risk relatives of patients with colorectal cancer. Within 9 months of randomization, 35% of individuals in the behavior change counseling intervention arm had undergone colonoscopy compared to 16% in the usual care arm (OR 2.83, *p* < 0.001).

In the current study, we adapted TeleCARE^6^ to evaluate the effect of a theory‐based behavioral intervention delivered by genetic counselors over the telephone on the uptake of prophylactic RRSO at 12 and 24 months by women with a *BRCA1* or *BRCA2* PV compared to women with a PV who received usual care.

## 2 METHODS

### 2.1 Study design and hypotheses

In this two‐arm, multi‐site randomized controlled trial (RCT), participants were randomized to the intervention arm or the usual care arm. Outcomes were measured at 12 months. For those in the control arm (usual care), the intervention was offered after completion of the 12 month outcome measures. All participants who had not undergone RRSO were contacted at 24 months to collect information on RRSO scheduling and/or completion.

We hypothesized that women who received the follow‐up theoretically‐guided behavioral telephone genetic counseling intervention would have higher uptake of RRSO at 1 year compared to women who received usual care. We also hypothesized that at 1 year after receiving the intervention, women would have lower decisional conflict and cancer‐related distress, and higher knowledge levels compared to women who received usual care. We also evaluated an exploratory aim recognizing that booking a gynecological consult, electing for surgery, and booking surgery may take longer than 1 year. For this exploratory aim, we hypothesized that 2 years after randomization, those who received the intervention would have higher uptake of RRSO compared to those who received usual care. The trial was approved by Research Ethics Boards at all participating centres.

### 2.2 Study participants

Eligible study participants included women enrolled in a multicenter, longitudinal study of *BRCA1* and *BRCA2* mutation carriers at 5 sites (Women's College Hospital; Grandriver Hospital; London Health Sciences Centre; Princess Margaret Hospital; Moffitt Cancer Center) in addition to self‐referrals through advertisements with commercial genetic testing laboratories and an online support group (Facing Our Risk of Cancer Empowered–FORCE).

Eligibility criteria included the following: (1) documented *BRCA1* or *BRCA2* PV; (2) age 35–70 years; (3) no previous RRSO; (4) no previous or current ovarian cancer; (4) at least 12 months since genetic testing or most recent contact by parent follow‐up study; (5) provided written consent to be re‐contacted for future research; and (6) could speak and understand English.

Women were excluded if: (1) currently receiving treatment for another cancer diagnosis (including breast cancer); (2) pregnant; (3) given birth in the last 6 months; or (4) were scheduled for or already had a surgical date for RRSO.

### 2.3 Screening and random assignment

Eligible participants were identified in the clinic databases at the participating centres and contacted by telephone. Written consent was obtained, and following the online collection of baseline data, all eligible, consented women were randomly allocated to either the intervention group (follow‐up telephone genetic counseling (FTGC) intervention) or the usual care group. Randomization was centrally controlled using a web‐based randomization service (www.randomize.net) with stratification based on age, gene (*BRCA1* versus *BRCA2*), and site of recruitment.

### 2.4 Data collection and primary outcome assessment

A research assistant blinded to group allocation contacted all participants by telephone at 12 months post‐randomization to collect outcome data (primary and secondary outcomes). After completion of follow‐up questionnaires, women in the usual care group were offered the follow‐up telephone genetic counseling intervention. At 2 years, all participants who had not undergone RRSO at year one were contacted over the telephone by the research assistant to collect data on uptake or scheduling of RRSO (self‐reported by participant).

### 2.5 Measures

#### 2.5.1 Primary outcome

*Uptake of RRSO*. The primary outcome was a yes/no response to uptake of RRSO (scheduled or completed) as identified by the *Follow‐up Questionnaire* administered at 12 months post‐randomization. This questionnaire included questions regarding any new diagnoses of cancer, uptake of risk‐reducing surgery, and intent for risk‐reducing surgery. If a participant answered no to having completed a RRSO, then they were asked if they had a RRSO scheduled. If yes, then they were classified as positive for uptake of RRSO.

#### 2.5.2 Secondary outcomes

##### Decisional conflict

Decisional conflict was assessed using the decisional conflict scale (DCS).^7^ The DCS measures a person's perception of the difficulty of making a decision (in this case about RRSO). The questionnaire consists of 16 items with response options ranging from 0 (strongly agree) to 4 (strongly disagree). Item responses are summed and scaled to create a total score ranging from 0 (no decisional conflict) to 100 (extremely high level of decisional conflict). Scores lower than 25 are associated with implementing decisions and scores exceeding 37.5 are associated with delay or feeling unsure about implementation.^8^ In addition to a total score, five subscale scores can be calculated representing uncertainty, information, values clarification, support and effective decision. In the trial sample, the alpha for the total scale was 0.95 or higher at all time points.

##### Cancer risk and prevention knowledge

Cancer risk and prevention knowledge was assessed using a knowledge index that was developed and tested by our group and used in previous studies involving women with *BRCA* pathogenic variants.^9^, ^10^ This index included items regarding risks of breast and ovarian cancer associated with having a *BRCA1/2* mutation, risk reductions associated with preventive options, and knowledge questions regarding each of the preventive options (risk‐reducing mastectomy, RRSO and chemoprevention). Scores were calculated by summing the number of correct answers, and total scores could range between 0 and 8.

##### Cancer‐related Distress

Cancer‐related Distress was assessed using the *Impact of Event Scale* (*IES*),^11^ a self‐report measure designed to measure current subjective distress about a specific stressor, in this case, risk of cancer. The scale consists of 15 items: 7 intrusion items and 8 avoidance items. Participants rate the frequency of intrusive and avoidant behaviors using a four‐point frequency scale (0 = not at all, 1 = rarely, 3 = sometimes, 5 = often). Scores range from 0 to 75, with separate intrusion and avoidance sub‐scales scores (with a possible range of 0–35 for intrusion, and 0–40 for avoidance). Lower scores depict lower levels of distress. In the trial sample, alpha was at least 0.91 and 0.88 for intrusion and avoidance, respectively, at all time points.

#### 2.5.3 Exploratory outcome

##### Uptake of RRSO at 2 years

An exploratory outcome was a yes/no response to uptake of RRSO (scheduled or completed) as identified by the follow‐up questionnaire administered at 2 years post‐randomization. Only participants who had not completed a RRSO at 12 months were contacted at 2 years. Similar to as described above for the primary outcome measure, women were classified as positive for uptake of RRSO if having undertaken or scheduled an appointment for the procedure.

### 2.6 Study intervention

Participants assigned to the intervention group received an intervention that was based on the TeleCARE intervention, that was shown to be effective at increasing uptake of colorectal screening in high‐risk relatives.^6^, ^12^ This intervention integrates the Extended Parallel Process Model (EPPM) and motivational interviewing. It is based on theoretical constructs that are hypothesized to motivate an individual with a *BRCA* mutation to undergo prophylactic RRSO (i.e., threat and efficacy perceptions and behavioral intentions).

This theory‐based behavioral telephone follow‐up genetic counseling (TFGC) intervention consists of several components:

- Part 1: Initial Assessment.
  All women randomized to the intervention completed the Risk Behavior Diagnosis Scale (RBDS)^13^ to assess perceptions about cancer risk and severity, response efficacy and self‐efficacy related to uptake of RRSO. The participant was mailed an educational brochure and tailored visual aids to be used during the telephone counseling session, which were tailored to each participant based on RBDS scores.
- Part 2: Telephone Follow‐up Genetic Counseling (TFGC) Intervention Delivery.
  The intervention delivered by genetic counselors was based on the EPPM, implementation intentions and motivational interviewing principles. To standardize the counseling sessions, all genetic counselors were trained and supervised by a motivational interviewing and EPPM expert. Each counselor completed 20 h of training time before delivering the intervention. In the month following the training, counselors submitted four practice tapes for coding and quality control, and participated in four weekly supervision sessions with the trainer. Tapes were coded using the Motivational Interviewing Treatment Integrity (MITI) coding system, a widely used measure of MI adherence.^14^ To ensure treatment fidelity, during the first 6 months of the intervention period, a random sample of tapes (one per counselor, per month) were coded, with summary scores provided to counselors on intervention delivery.

One trained genetic counselor from each participating cancer genetics clinic delivered the intervention to ensure that participants continued to have ongoing relationships with their original cancer genetics clinic. The intervention incorporated risk communication and behavior change approaches designed to: (1) raise awareness of threats about *BRCA*‐associated breast and ovarian cancers; (2) raise awareness of the benefits of RRSO; and (3) address barriers to help increase motivation and self‐efficacy to elect for the preventive surgery. The counselor tailored the counseling session based on the participants' RBDS scores. As a component of the counseling, participants were asked if they would like a referral to a local gynecological surgeon to discuss RRSO. A tailored letter was sent to the participant describing the current recommendations and details of the referral if appropriate.

### 2.7 Usual care

Women allocated to the usual care arm had access to standard post‐genetic testing care provided to all women identified with a *BRCA* PV (which includes referral to appropriate specialists). After the assessment at 12‐month post‐randomization, women in usual care were offered the intervention.

### 2.8 Statistical analysis

The analyses used an intent to treat (ITT) approach and included all eligible participants who were randomly assigned to a treatment arm. Balance in background clinical and demographic covariates was assessed across arms using *t*‐tests and Chi‐squared tests or Fisher's Exact tests as required. The primary outcome of the proportion of participants who received or scheduled a RRSO by the 1‐year follow‐up was assessed using logistic regression. For those with an unknown outcome due to loss to follow‐up, negative and multiple imputations methods were undertaken and compared to the known outcomes analysis. Negative imputation assumes that if no record of the outcome is observed then the procedure was not scheduled or undertaken. Multiple imputation (MI) used information on verified RRSO by year 1 and background variables, including age, education level, income level, presence of children, breast cancer diagnosis, mother had ovarian cancer (yes/no), annual ovarian screening (yes/no) to replace missing outcome values. Using the fully conditional method (FCS), which implements chained equations, 20 models were imputed.

The differences by treatment arm in secondary psychosocial outcomes were assessed using Linear Mixed Models (LMM), which upholds the ITT principle by using all available data. This method is comparable to ANCOVA with MI but provides more power, especially as the amount of missing data increases.^15^ An unstructured covariance matrix was used with the Kenward–Rodger approximation method; the latter tends of perform better under small sample conditions.^16^ Least squares means (LSM) derived from the LMM are presented with 95% confidence intervals. Raw pre‐ and post‐test scores by treatment arm are presented for descriptive purposes.

A post hoc exploratory analysis was undertaken to determine and compare across arms the proportion of participants who received or scheduled a RRSO by 2 years post‐randomization and the incidence of received or scheduled RRSO between 1 and 2 years post‐randomization. For the latter calculation, only those who continued to be “at risk” were included in the analysis (i.e., those who had/scheduled a RRSO or who dropped out by year 1 were removed). Similar methods for modeling and dealing with missing data were used as for the primary outcome analysis.

All analyses were undertaken using SAS (version 9.4; SAS Institute). To evaluate the primary outcomes, alpha was set at 0.05. For secondary outcomes, to accommodate multiple testing, the alpha was set to 0.005 using a Bonferroni correction.

### 2.9 Sample size

In the previous follow‐up telephone‐based genetic counseling intervention by Kinney and colleagues, uptake of colonoscopy was 15% in the usual care group and 35% in the intervention group. Since uptake of RRSO was not expected to be as high as colonoscopy (which is a screening intervention rather than a prophylactic surgical intervention), we based our sample size calculation on the ability to detect a slightly smaller difference of approximately 15%. Sample sizes of 121 in the intervention arm and 121 in the usual care arm were required to detect this difference with 80% power, using a two‐sided *Z* test with pooled variance and alpha set at 0.05. To account for 20% loss to follow‐up, a total sample size of 304 (152 per group) was targeted. Due to recruitment challenges (including lower than expected number of eligible participants at the study sites), the required sample size was not achieved. With the sample size recruited, we had 46% power to detect a difference of 15% at 1 year of follow‐up.

## 3 RESULTS

### 3.1 Study cohort

**FIGURE 1:**
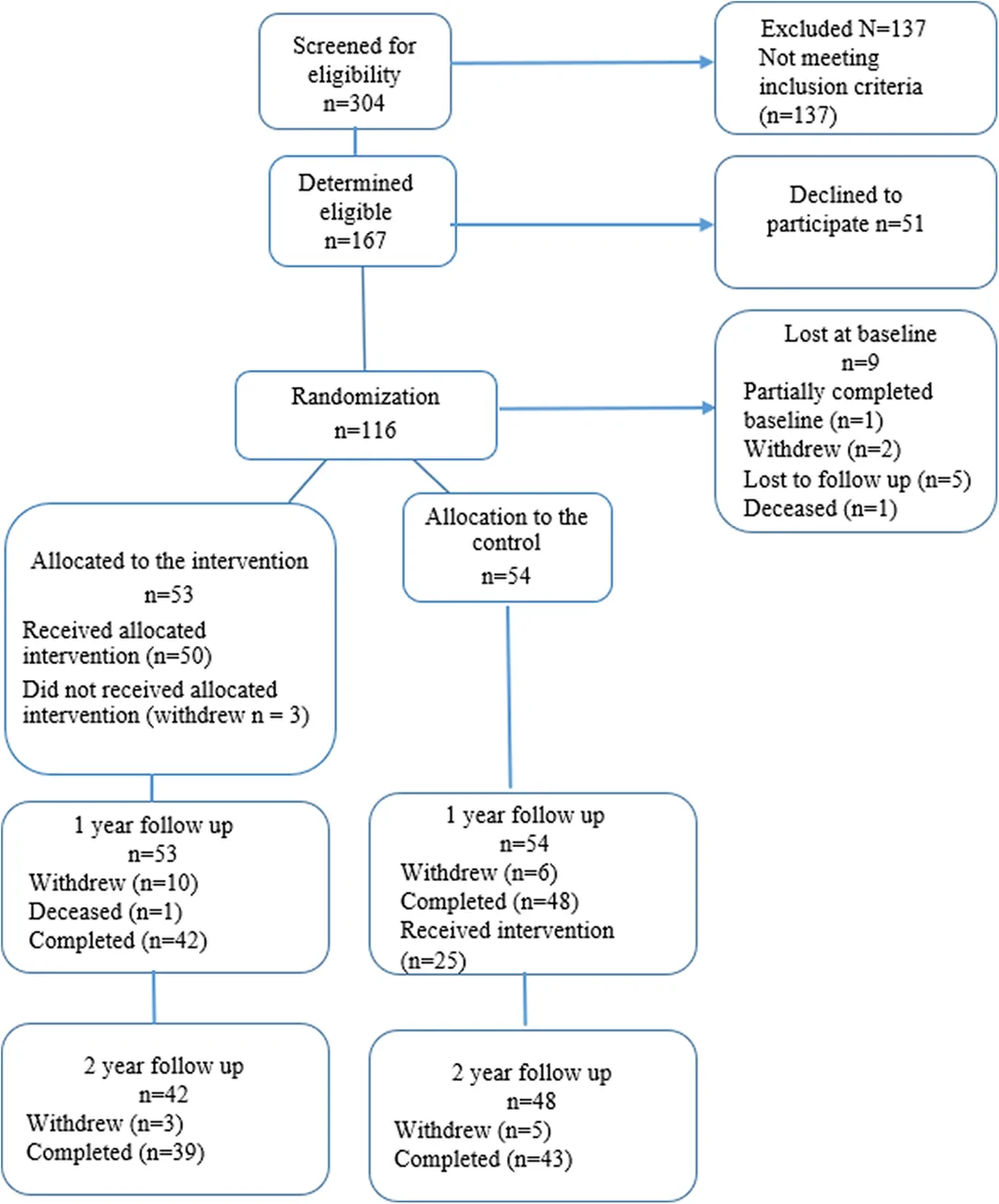
CONSORT flow chart.

The CONSORT flowchart is shown in Figure 1. A total of 167 women were deemed eligible to participate in the study, of which 116 were enrolled. Nine individuals were lost between enrollment and baseline due to withdrawal, loss to follow up or death, resulting in a total of 107 participants who were included in the analysis in one of the two treatment arms: 53 in the intervention arm and 54 in the usual care arm. Overall, 94% of those in the intervention arm received the behavioral intervention delivered by genetic counselors.

A total of 107 participants were included in the outcome analysis. For 17 of these participants, no outcome data were available at 1 year, resulting in a retention rate of 84.1%; there was no significant difference in retention by treatment arm (*p* = 0.46). Those with follow‐up data at 1 year and those without did not differ significantly on any key demographic or clinical background variables.

Table 1 shows the baseline demographic and clinical characteristics by treatment arm. The mean age of participants was 45.2 (SD = 8.5) years. Overall, 57% of participants had a *BRCA1* mutation, and 43% had a *BRCA2* mutation. A total of 63% of participants had previously consulted with a surgeon about RRSO and 17% had a mother with ovarian cancer.

**TABLE 1:** Baseline demographic & clinical characteristics by treatment arm for intent‐to‐treat analysis.

	Overall (*n* = 107)	Intervention (*n* = 53)	Control (*n* = 54)	*p* value
Age, mean (SD)	45.2 (8.5)	44.9 (8.4)	45.5 (8.7)	0.68
Country				
Canada	70 (66.0)	36 (69.2)	34 (63.0)	0.50
US	36 (34.0)	16 (30.8)	20 (37.0)	
Uninsured (US only)	4 (11.1)	1 (6.3)	3 (15.0)	0.61
Education				
No post‐secondary degree/diploma	22 (20.6)	12 (22.6)	10 (18.5)	0.45
College or trade school degree/diploma	17 (15.9)	11 (20.8)	6 (11.1)	
University undergraduate degree	35 (32.7)	16 (30.2)	19 (35.2)	
University graduate degree	33 (30.8)	14 (26.4)	19 (35.2)	
Marital status				
Single	19 (17.9)	7 (13.5)	12 (22.2)	0.16
Married/common‐law	76 (71.9)	37 (69.8)	39 (72.2)	
Divorced/separated	11 (10.4)	8 (15.1)	3 (5.6)	
Have children	83 (77.6)	46 (86.8)	37 (68.5)	0.02
Planning to have children	11 (10.3)	7 (13.2)	4 (7.4)	0.32
Household income				
<$50,000	10 (11.6)	4 (9.1)	6 (14.3)	0.30
$50,000–$80,000	18 (20.9)	12 (27.3)	6 (14.3)	
>$80,000	58 (67.4)	28 (63.6)	30 (71.4)	
Employment status				
Full time	69 (64.5)	37 (69.8)	32 (59.3)	0.42
Part time	19 (17.8)	7 (13.2)	12 (22.2)	
Not working for pay	19 (17.8)	9 (17.0)	10 (18.5)	
Cultural group				
South Asian	2 (1.9)	1 (1.9)	1 (1.9)	0.84
Asian or Pacific Islander	3 (2.8)	1 (1.9)	2 (3.7)	
Black non‐Hispanic	3 (2.8)	1 (1.9)	2 (3.7)	
White non‐Hispanic	78 (72.9)	41 (77.4)	37 (68.5)	
White hispanic	5 (4.7)	1 (1.9)	4 (7.4)	
Indigenous North American	5 (4.7)	3 (5.7)	2 (3.7)	
Other	11 (10.3)	5 (9.4)	6 (11.1)	
BRCA mutation				
BRCA 1	61 (57.0)	29 (54.7)	32 (59.3)	0.64
BRCA 2	46 (43.0)	24 (45.3)	22 (40.7)	
Diagnosed with breast cancer	42 (39.3)	25 (47.2)	17 (31.5)	0.10
Consulted surgeon about PO	67 (62.6)	33 (62.3)	34 (63.0)	0.94
Mother with ovarian cancer	18 (16.8)	10 (18.0)	8 (14.8)	0.58
Sister with ovarian cancer	5 (4.7)	1 (1.9)	4 (7.4)	0.15
Undergo annual ovarian screening	62 (57.9)	30 (56.6)	32 (59.3)	0.78
CA 125	46 (43.0)	21 (39.6)	25 (46.3)	0.49
Transvaginal ultrasound	60 (56.1)	29 (54.7)	31 (57.4)	0.80

There were no differences in clinical characteristics between arms. A single demographic variable was different across arms: a higher proportion of individuals in the intervention arm had children compared to the usual care arm (*p* = 0.024); however, there was no difference across arms in intention to have future children (*p* = 0.323). Because having children versus not having children may affect decisions regarding undertaking a RRSO, this variable was adjusted for in the modeling. As this was not a planned adjustment, both unadjusted and adjusted models are presented.

#### 3.1.1 Primary outcome

There was no difference in the proportion of women who scheduled or underwent a RRSO by the 1 year follow‐up. For those with a known outcome, the raw proportions of RRSO were 28.6% in the intervention arm and 22.9% in the usual care arm (*p* = 0.54). The expected difference of 15% between arms was not found at 1 year. Similar results were observed across imputation methods and whether adjustment for baseline imbalances were undertaken (Table 2).

**TABLE 2:** Intervention effect on rRSO booking/uptake using logistic regression at 1 year follow‐up based on different methods of dealing with missing data.

Outcome	Odds of PO in intervention v. control	95% CI	*p* value	Intervention		Control	
				No.	%	No.	%
Outcome known	1.35	0.52–3.48	0.54	12 of 42	28.6	11 of 48	22.9
Outcome known ‐ adjusted^a^	1.29	0.49–3.43	0.61				
Imputation							
Negative^b^	1.14	0.45–2.88	0.78	12 of 53	22.6	11 of 54	20.4
Negative–adjusted	1.14	0.44–2.94	0.79				
Multiple^c^	1.46	0.55–3.82	0.45	16 of 53	30.2	12 of 54	22
Multiple–adjusted	1.41	0.53–3.78	0.49				

^a^ Adjusted for having children.

^b^ Negative outcome imputation treated unknown PO as no PO.

^c^ Average number of PO/bookings based on twenty imputation sets.

#### 3.1.2 Secondary outcomes

**FIGURE 2:**
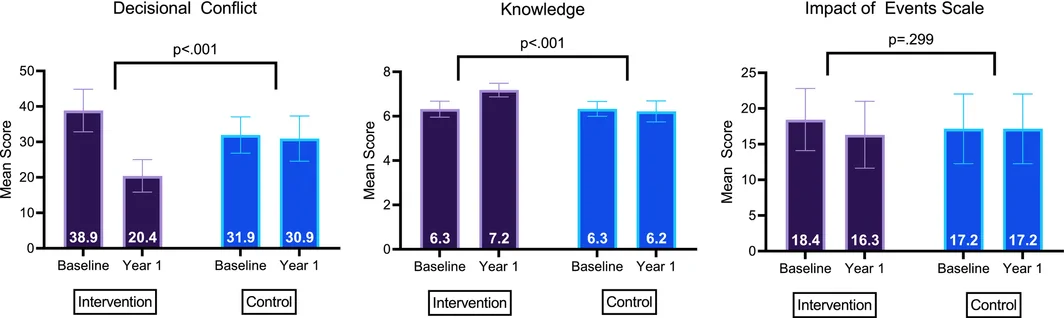
Comparison of raw scores of secondary measures between treatment arms at pre‐ and post‐test. *p* value is derived ANOVCA.

Figure 2 shows the raw scores of the secondary measures by treatment arm at pre‐ and post‐test for descriptive purposes. Table 3 shows the ITT analyses, which are the focus of the results presented below.

**TABLE 3:** Differences in secondary outcomes at follow up by treatment arm using all available data in linear mixed modeling.

	Intervention			Control			*p*‐value
	Least squares means	95% confidence interval		Least squares means	95% confidence interval		
DCS–Total	17.5	11.2	23.8	30.6	25	36.2	<0.001
DCS–Uncertainty	28.7	20.5	37	43.1	36	50.2	<0.001
DCS–Information	12.8	5.7	19.9	23.4	7.3	29.6	0.04
DCS–Values clarification	15.5	8.6	22.4	27.1	21.1	33.1	0.006
DCS–Support	14.5	6.9	22.2	25.8	19.1	32.4	0.004
DCS–Effective	17.7	10.4	25.1	29.3	23	35.7	0.002
IES–Total	15.1	9.5	20.7	17.2	12.4	22.1	0.31
IES–Avoid	7.6	4.5	10.8	9.4	6.7	12.2	0.41
IES–Intrusion	7.5	4.7	10.3	7.8	5.4	10.2	0.33
Knowledge score	7	6.6	7.5	6.2	5.8	6.6	<0.001

*Note*: Outcomes are adjusted for having children.

Abbreviations: DCS, Decisional Conflict Scale; IES, Impact of Events Scale.

##### Decisional conflict

As shown by the linear mixed modeling, the intervention arm had a significantly lower mean decisional conflict at year 1 (LSM =17.5; 95% CI = 11.2–23.8) compared to the usual care arm (LSM = 30.6; 95% CI 25.0–36.2; *p*_interaction_ <0.001). Using the adjusted alpha of 0.005, three decisional conflict subscales were also significantly lower in the intervention arm at follow up: uncertainty, support and effective decision (Table 3). The unadjusted estimates were highly consistent with the adjusted estimates and can be found in the Supplemental File.

##### Cancer risk & prevention knowledge

The raw baseline knowledge score was 6.3 (SD = 1.3) for the intervention arm and 6.3 (SD = 1.2) for the usual care arm. The linear mixed modeling found that the intervention arm had a significantly higher mean knowledge score at one‐year (LSM = 7.0, 95% CI = 6.6–7.5) compared to the usual care arm (LSM = 6.2; 95% CI = 5.8–6.6; *p*_interaction_ <0.001).

##### Cancer‐related distress

The raw baseline IES score was 18.4 (SD = 15.8) for the intervention arm and 17.1 (SD = 17.9) for the usual care arm. Based on the linear mixed modeling, the intervention arm had a non‐significantly lower mean total IES score at year 1 (LSM = 15.1, 95% CI = 9.5–20.7) compared to the usual care arm (LSM = 17.2, 95% CI = 12.4–22.1; *p*_interaction_ = 0.32). Neither IES subscale was significantly different by treatment arm (Table 3).

#### 3.1.3 Exploratory outcome

Based on the original arm allocation, the proportion of women who scheduled or underwent a RRSO by the year 2 follow‐up was higher in the intervention arm compared to the usual care arm based on analysis of known outcomes (*p* = 0.05). All participants who reported that they had scheduled a RRSO at year 1 had undergone RRSO by year 2. The raw proportions of RRSO at year 2 were 53.9% in the intervention arm and 32.6% in the control group, exceeding the 15% expected difference (Table 4). Results were similar across methods of dealing with missing data. When looking at the incidence of RRSO between year 1 and year 2, there was a significant difference between arms, with the intervention arm having a higher proportion. The raw proportions of RRSO between year 1 and year 2 were 33.3% in the intervention arm and 9.4% in the usual care arm (*p* = 0.03). The significant difference was found with adjustment and across both methods of dealing with missing data. Of those in the usual care arm with a known outcome who received the intervention after year 1 (and did not have a RRSO by year 1), 2 received/scheduled a RRSO out of 22. This is compared to 1 out of 10 with a known outcome in the usual care arm who never received the intervention after year 1 (*p* = 0.94).

**TABLE 4:** Intervention effect on BSO booking/uptake using logistic regression between year 1 and year 2 follow‐up based on different methods of dealing with missing data.

	Cumulative incidence across 2 years							Incidence from year 1 to year 2^a^						
	Odds of RRSO in intervention v. control	95% CI	*p* value	Intervention		Control					Intervention		Control	
				No.	%	No.	%	Odds of RRSO in intervention v. control	95% CI	*p* value	No.	%	No.	%
Outcome known	2.42	0.99–5.92	0.05	21 of 39	53.9	14 of 43	32.6	4.83	1.15–20.3	0.03	9 of 27	33.3	3 of 32	9.4
Outcome known ‐ adjusted^b^	2.4	0.96–6.02	0.06					4.85	1.15–20.5	0.03				
Imputation														
Negative^c^	1.88	0.83–4.26	0.13	21 of 53	39.6	14 of 54	25.9	4.86	1.18–20.0	0.03	9 of 30	30	3 of 37	8.1
Negative^c^−adjusted^b^	1.96	0.84–4.55	0.12					4.95	1.18–20.7	0.03				
Multiple^d^	2.2	0.92–5.18	0.08	29 of 53	54.7	19 of 54	35.2	4.29	1.04–17.6	0.04	10 of 30	33.3	4 of 37	10.8
Multiple^d^−adjusted^b^	2.11	0.87–5.12	0.1					4.28	1.02–17.9	0.05				

^a^ Those who obtained or scheduled a BSO or dropped out by year 1 are removed from this analysis.

^b^ Adjusted for having children.

^c^ Negative outcome imputation treated unknown PO as no PO.

^d^ Average number of PO/bookings based on 20 imputation sets.

## 4 DISCUSSION

For women with a *BRCA1* or *BRCA2* PV, risk‐reducing salpingo‐oophorectomy (RRSO) between the ages of 35 and 45 years is recommended in international guidelines. However, uptake is not ideal.^5^ In the current study we evaluated a theory‐based behavioral intervention delivered by genetic counselors that integrated the Extended Parallel Process Model and motivational interviewing focusing on RRSO for women with a *BRCA* PV who had not undergone the recommended preventive surgery. After 1 year, there was no significant difference in uptake of RRSO in women who received the intervention compared to those who received usual care. After 1 year, women who received the intervention had significantly lower levels of decisional conflict and higher knowledge levels compared to those who received usual care. In an exploratory analysis, women who received the intervention had significantly greater uptake of RRSO at 2 years compared to those who received usual care.

Pre‐menopausal removal of the ovaries results in abrupt surgical menopause and menopausal symptoms, including more vaginal dryness and dyspareunia and less pleasure and satisfaction during sexual activity.^17^, ^18^, ^19^, ^20^, ^21^, ^22^ Physical and mental health‐related quality of life is similar to that of population controls before and after surgery.^23^ As a result of these possible side‐effects, some women struggle with the decision to have RRSO. Uncertainty about the course of action given competing priorities such as risk, loss, regret or challenges to personal life values is referred to as decisional conflict.^24^ In lay terms, decisional conflict is indicative of the level of comfort an individual feels in making a decision.^25^ Decisional conflict scores are highly predictive of the extent to which individuals make health‐related decisions.^26^

In the current study, women had elevated levels of decisional conflict related to RRSO at baseline. These elevated levels of decisional conflict were observed again at 1 year for women randomized to usual care. However, for women who received the intervention, decisional conflict was significantly lower at 1 year. Women in the intervention arm went from scores that suggested that a decision about RRSO would not be made, to a mean score suggesting that a decision would be made. This suggests that the behavioral intervention may have targeted domains specific to decisional conflict, including knowledge, harms and benefits of options, and support.^25^

Knowledge is a modifiable decisional need that contributes to decisional conflict.^24^ In the current study, knowledge was significantly higher at one‐year for women who received the intervention compared to those who received usual care. The intervention included educational components about cancer risk and severity. Key components of the intervention were based on the Extended Parallel Process Model (EPPM).^27^ According to the EPPM, people who believe that the recommended action (e.g., RRSO) is effective at preventing the threat (e.g., breast and ovarian cancer, death) and have confidence in their ability to implement the action (high self‐efficacy) are better able to deal with the threat by using danger control strategies. In the current study, this was accomplished through acknowledging and following through with the recommended action (RRSO).

At one‐year post‐randomization, there were no differences in RRSO uptake between the intervention and usual care groups (28.6% vs. 22.9%; *p* = 0.54). However, in an exploratory analysis there was a significant difference between the groups at 2 years post‐randomization. The raw proportions of RRSO were 53.9% for those who received the intervention and 32.6% for those who received usual care. This unexpected finding surpassed the 15% expected difference in uptake between the two groups based on the TeleCARE intervention study results. Some women in the control arm did elect to receive the intervention after 12 months, and there was no difference in uptake of RRSO by 24 months between those who received the intervention and those who did not. Although this study was not designed to measure the primary outcome at 2 years, these data suggests that future evaluations of uptake should incorporate longer follow‐up to ensure that women have adequate time to be referred for a gynecological consult and book this elective surgery. Wait times for consultations and surgery vary by location and may have contributed to lower than expected uptake rates of RRSO at 1 year.

Most women who learn that they have a *BRCA1* or *BRCA2* PV do not receive long‐term genetic counseling follow‐up. This may contribute to the less than ideal uptake rates of RRSO in this population. In the current study, we demonstrated that a follow‐up theory‐based behavioral intervention delivered by genetic counselors increased RRSO among women who had not elected for the procedures after receiving their initial positive genetic test results. However, this may require additional genetic counseling resources which may be challenging in the current clinical environment. Additional follow‐up interventions require evaluation, including provision of the intervention by other healthcare providers including specially trained nurses or other healthcare professionals.

There are several strengths to this study including the rigorous methodology employed in the randomized controlled trial, including stratification by site of referral. We recruited women from throughout North America using a variety of sources, including self‐referral which contributed to the generalizability of our findings. We used a theoretically‐derived intervention with relatively rigorous supervision and quality control.

There are limitations to this study, including the small sample size that did not meet the planned sample size requirements (not powered for primary outcome). We still observed significant differences between groups in secondary outcomes, including decisional conflict and knowledge. In addition, we did observe significant differences in RRSO uptake after 2 years between those who received the intervention compared to those who received usual care, which could have been due to wait times for consulations and surgery. In addition, the women included in this study self‐selected to be involved in a research study and may not be representative of the population of women with a *BRCA1* or *BRCA2* mutation. Finally, the study population was primarily non‐Hispanic White thereby limiting the generalizability of our findings.

Overall, this study provides evidence that a theory‐based behavioral intervention delivered by genetic counselors to women with a *BRCA* PV who had not elected for the recommended RRSO was effective at reducing decisional conflict about having a RRSO, and increasing knowledge about the effectiveness of cancer risk reduction options in women with a *BRCA1* or *BRCA2* PV. There was no statistical difference in uptake of RRSO after 1 year between those who received the intervention and those who received usual care. Further research should be done to explore our exploratory finding that after 2 years significantly more women had a RRSO in the intervention group compared to those who received usual care. Our results suggest that women with a *BRCA1* or *BRCA2* PV who have not elected for a recommended RRSO may benefit from theory‐based behavioral interventions to increase uptake of RRSO.

## CLINICAL TRIAL REGISTRATION NUMBER

NCT02225015.

## AUTHOR CONTRIBUTIONS

**Kelly A. Metcalfe:** Conceptualization (lead); data curation (equal); formal analysis (equal); funding acquisition (lead); investigation (lead); writing – original draft (lead). **Tuya Pal:** Data curation (equal); investigation (equal); writing – review and editing (equal). **Steven Narod:** Data curation (equal); investigation (equal); writing – review and editing (equal). **Susan Armel:** Data curation (equal); writing – review and editing (equal). **Salma Shickh:** Data curation (equal); investigation (equal); writing – review and editing (equal). **Kathleen Buckley:** Data curation (equal); writing – review and editing (equal). **Scott Walters:** Investigation (equal); methodology (equal); writing – review and editing (equal). **Sarah Brennenstuhl:** Formal analysis (lead); writing – review and editing (equal). **Anita Kinney:** Conceptualization (equal); investigation (equal); methodology (equal); writing – review and editing (equal).

## FUNDING INFORMATION

This study was funded by a CIHR grant awarded to KM.

## CONFLICT OF INTEREST STATEMENT

The authors declare that there is no conflict of interest.

## Supporting information


Table S1.

## Data Availability

Data sharing is not applicable to this article as no new data were created or analyzed in this study.
