# Supplementary material for: Theory‐based behavior change intervention to increase uptake of risk‐reducing salpingo‐oophorectomy in women with a BRCA1 or BRCA2 pathogenic variant: The PREVENT randomized controlled trial
Source: Cancer Med. 2023 Aug 21;12(17):18246–57. doi: 10.1002/cam4.6417 (PMC10524042; doi:10.1002/cam4.6417)
Supplement: Supplementary file 1 — Table S1. [file CAM4-12-18246-s001.pdf]

Supplemental Table. Differences in Secondary Outcomes at Follow up by Treatment Arm Using all Available Data in Linear Mixed Modeling without Adjustment for Having Children

	Intervention			Control			P-value*
	Least Squares Means	95% Confidence Interval		Least Squares Means	95% Confidence Interval		
DCS - Total	20	14.2	25.8	31.9	26.5	37.4	<.001
DCS - Uncertainty	30.9	23.4	38.4	44.1	37.1	51.1	<.001
DCS - Information	16.4	9.9	23	25.1	19	31.2	0.0361
DCS - Values clarification	18.2	11.8	24.6	28.3	22.4	34.2	0.0064
DCS - Support	16.1	8.9	23.2	26.5	19.9	33	0.0036
DCS - Effective	19.7	12.9	26.5	30.2	24	36.5	0.0018
IES - Total	16.1	11.2	21.1	17.7	13	22.5	0.3165
IES - Avoid	8.5	5.7	11.3	9.8	7.1	12.5	0.4162
IES - Intrustion	7.7	5.3	10.1	7.9	5.6	10.2	0.3308
Knowledge score	7	6.5	7.4	6.1	5.8	6.5	<.001

DCS, Decisional Conflict Scale; IES, Impact of Events Scale
